# Identification of an exonic splicing silencer in exon 6A of the human VEGF gene

**DOI:** 10.1186/1471-2199-10-103

**Published:** 2009-11-17

**Authors:** Rui Wang, Ronald G Crystal, Neil R Hackett

**Affiliations:** 1 Department of Genetic Medicine, Weill Cornell Medical College, New York, NY, USA

## Abstract

**Background:**

The different isoforms of vascular endothelial growth factor (VEGF) play diverse roles in vascular growth, structure and function. Alternative splicing of the VEGF gene results in the expression of three abundant isoforms: VEGF121, VEGF165 and VEGF189. The mRNA for VEGF189 contains the alternatively spliced exon 6A whereas the mRNA for VEGF165 lacks this exon. The objective of this study was to identify the *cis *elements that control utilization of exon 6A. A reporter minigene was constructed (pGFP-E6A) containing the coding sequence for GFP whose translation was dependent on faithful splicing for removal of the VEGF exon 6A. To identify *cis*-acting splicing elements, sequential deletions were made across exon 6A in the pGFP-E6A plasmid.

**Results:**

A candidate *cis*-acting exonic splicing silencer (ESS) comprising nucleotides 22-30 of exon 6A sequence was identified corresponding to the a silencer consensus sequence of AAGGGG. The function of this sequence as an ESS was confirmed *in vivo *both in the context of the reporter minigene as a plasmid and in the context of a longer minigene with VEGF exon 6A in its native context in an adenoviral gene transfer vector. Further mutagenesis studies resulted in the identification of the second G residue of the putative ESS as the most critical for function.

**Conclusion:**

This work establishes the identity of *cis *sequences that regulate alternative VEGF splicing and dictate the relative expression levels of VEGF isoforms.

## Background

Angiogenesis is a critical component of many physiological and pathological processes such as tissue repair and tumor growth. VEGF is the most powerful angiogenic factor mediating developmental, physiological and pathological angiogenesis [1-3]. VEGF gene expression is a complex process with regulation at the level of transcription, mRNA stability and translation [4-10]. Through alternate splicing, at least eight different isoforms of VEGF are formed, comprising VEGF206, 189, 183, 165, 148, 145, 121, and an inhibitory isoforms, 165b [11-13]. Some isoforms such as VEGF183 and 206 are expressed in a cell and tissue restricted manner and the mechanisms by which they are selectively spliced is unknown [14-16]. But among the isoforms, VEGF189, 165 and 121 are the most abundant in most tissues [17,18]. VEGF189 mRNA is relatively abundant in mouse lung and heart, but VEGF165 mRNA has the highest level of expression in most other mouse tissues [17-20]. These two isoforms differ by the presence or absence of exon 6A which encodes the critical amino acids that confer differences in biological properties between VEGF165 andVEGF189 [11,20]. The utilization of exon 6A presumably involves many factors such as *cis*-acting RNA sequences within the exons and flanking introns, and interactions with components of the basal and alternate splicing machinery and auxiliary regulatory factors which transiently co-assemble with the spliceosome.

The biological characteristics of the different VEGF isoforms are strikingly different with VEGF121 being soluble but the longer isoforms, especially VEGF189, binding to heparan in the extracellular matrix at the locations where it is synthesized [20]. Cleavage of matrix associated VEGF189 by proteases such as plasmin is critical for its biological activity [20]. VEGF isoforms have different affinities for the VEGF receptors [VEGFR1 (flt1), VEGFR2 (KDR/flk1) and VEGFR3 (neuropilin)] and may play distinct roles in vascular development and diseases such as cancer growth and metastasis [19,21,22]. VEGF165 is able to bind to VEGFR1, VEGFR2 as well as neuropilin-1; VEGF121 binds to VEGFR1 and VEGFR2, but not neuropilin-1, and VEGF189 binds to VEGFR1 in its native form and binds to both VEGFR1 and VEGFR2 in its cleaved form [3,23]. In general, studies on tumors which overexpress VEGF121, VEGF165 or VEGF189 show that the longer isoforms, especially VEGF189 or the mouse equivalent VEGF188, are more effective in supporting tumor growth and establishing xenografts [24-26]. The enhanced *in vivo *growth of tumors expressing VEGF189 can be partly explained by the cell-associated features of VEGF189 and its high potential for induction of local angiogenesis and tumor growth in cancer inductive microenvironments [27]. The different biologic characteristics of the VEGF isoforms are also relevant to VEGF-mediated therapeutic angiogenesis to treat disorders such as coronary artery disease or peripheral vascular disease. In our studies of angiogenic gene transfer, we discovered that simultaneous expression of multiple VEGF isoforms resulted in a more potent angiogenic signal than a single isoform, presumably due to the overlapping biochemical characteristics [28]. Other studies showed that over-expression of VEGF189 provided a more favorable safety profile than VEGF165 [29,30].

Based on these considerations, the objective of this study was to test the hypothesis that *cis *sequences can be identified within the VEGF exon6A that control utilization of exon 6A and promote or suppress production of VEGF189. Using a reporter gene and *in vitro *gene transfer assays, a *cis *acting element within exon 6A that significantly affected the balance between VEGF189 and VEGF165 was identified. Deletion and point mutations in a putative exonic splicing silencer were created that markedly enhanced the utilization of exon6A *in vitro*. These mutations were then moved back into the context of the VEGF gene and shown to have a similar effect on the splicing of the VEGF gene, providing a basis for better understanding VEGF splicing and cell-specific expression of VEGF isoforms.

## Results

### Generation of GFP Reporter Minigene

**Figure 1:**
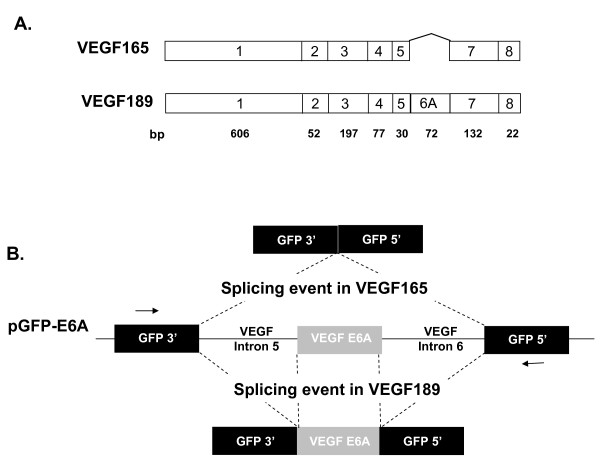
**Splicing of endogenous VEGF gene and design of GFP reporter minigene for assessing VEGF exon 6A splicing**. **A**. Exon utilization in VEGF165 and VEGF189. Both VEGF165 and VEGF189 use exons 1 through 5 plus exon 7 and 8. The mRNA for VEGF189 also contains the alternatively spliced exon 6A. **B**. Design of the GFP reporter minigene for assessment of VEGF exon 6A usage. The minigene consists of a GFP gene driven by cytomegalovirus (CMV) promoter which is interrupted by a shortened version of VEGF intron 5, exon 6A and a shortened version of intron 6. Detailed description of the components is given in Methods. There are two possible splicing reactions for the transcripts of pGFP-E6A. If the splicing pattern is like that in VEGF189, exon 6A is included so as to interrupt the GFP reading frame. If the splicing pattern is similar to that in VEGF165, exon 6A is excluded and a complete GFP open reading frame is generated giving rise to active GFP protein. The arrows indicated the position of primers used for amplification of spliced mRNA products.

A minigene reporter system, pGFP-E6A, was developed in which the splicing of the human VEGF exon 6A could readily be monitored. The minigene consisted of a GFP expression plasmid in which the GFP gene sequence was interrupted by a shortened version of intron 5, the complete exon 6A and a shortened version of intron 6 all from the human VEGF gene (Figure 1). Upon transcription, there could be two possible splicing events. If the splicing pathway corresponds to that of VEGF189, exon 6A will be included in the final mRNA and the GFP open reading frame will be interrupted. Conversely, if the pGFP-E6A transcript follows the VEGF165 splicing pathway, VEGF exon 6A is excluded and an intact open reading frame for GFP is obtained with expression of GFP.

**Figure 2:**
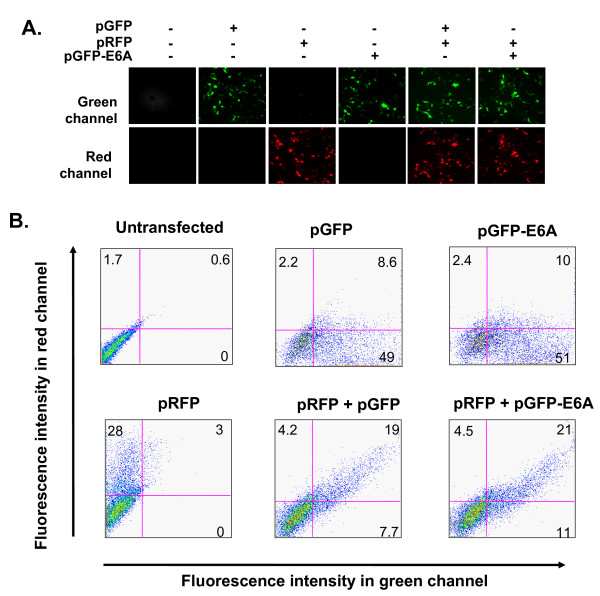
**Verification of splicing pattern ofrom transfected pGFP-E6A reporter minigene**. **A**. HEK293 cells were transfected with combinations of pGFP, pRFP and pGFP-E6A as shown above each pair of photographs. After 48 hr, the fluorescence was assessed in the green and red channels. All photographs taken with the same exposure to compare intensities. **B**. Flow cytometry of HEK293 cells 48 hr post transfection assessing fluorescent intensity in red and green channels. The percentages of fluorescent cells are shown in each quadrant.

Human embryonic kidney 293 cells (HEK293) were used to assess splicing of the transfected reporter minigene. The HEK293 cells were transfected with pGFP-E6A and/or control plasmids expressing just GFP or RFP and the cells were subsequently examined by fluorescent microscopy and flow cytometry (Figure 2). After transfection of the pGFP and pRFP control plasmids, cells of the expected color were observed by fluorescent microscopy and when both plasmids were cotransfected, cells with fluorescence in red and green channels were observed (Figure 2A). When the pGFP-E6A plasmid was transfected, green fluorescence was observed by microscopy comparable in intensity to that seen in cells transfected with pGFP. Cotransfection with pRFP allowed comparable signal to be seen in both channels suggesting the pGFP-E6A plasmid is efficiently spliced to form the intact open reading frame of GFP, i.e., it was spliced in the same pattern as inVEGF165. Flow cytometry was used to quantify the fluorescence (Figure 2B). The frequency of GFP, RFP double positive cells was similar (19%) when the pGFP control plasmid was contransfected with pRFP as when pGFP-E6A was contransfected with pRFP (21%). We conclude that the VEGF 165 splicing pathway is predominant in this cell type as reflected in the splicing of both the endogenous VEGF gene and the transfected reporter minigene.

**Figure 3:**
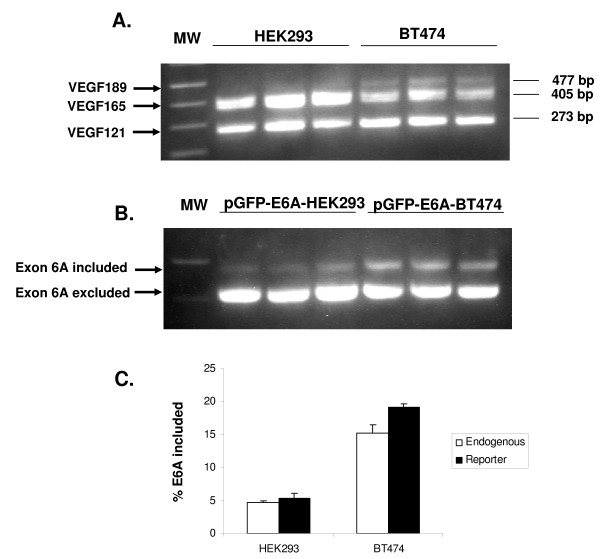
**Splicing profile of endogenous VEGF gene andtransfected with pGFP-E6A plasmids in HEK293 and BT474 cells**. **A**. mRNA for endogenous human VEGF isoforms were amplified using specific primers on mRNA extracted from of HEK293 and BT474 cells. The expected sizes of the products are indicated on the right. **B**. HEK293 and BT474 cells in triplicate were transfected with pGFP-E6A. After 48 hr, total RNA were assessed using RT-PCR. The anticipated positions of the products from the splicing events with inclusion or exclusion of exon 6A are indicated by arrows. **C**. Comparison of Exon 6A inclusion rate for endogenous and transfected reporter minigene between HEK293 cells and BT474 cells. The average ± standard error for three experiments is shown.

To confirm that the pGFP-E6A plasmid faithfully reports the splicing pattern of the endogenous VEGF gene, the splicing of endogenous VEGF gene and transfected pGFP-E6A was compared in HEK293 and the BT474 cell line. BT474 cells have been reported to express a high level of VEGF189 [31]. In BT474, the average rate of endogenous VEGF E6A inclusion in 3 independent experiments was 15.2 ± 1.3% compared with 4.7 ± 0.3% in HEK293 cells (Figure 3). For pGFP-E6A plasmid transfected cells, the average E6A inclusion rate in BT474 was 19.1 ± 0.5% compared with 5.3 ± 0.8% in HEK293 cells (Figure 3). Thus, exon 6A utilization for endogenous VEGF gene and the reporter genes in different cells types were comparable.

### Mapping of the *cis*-acting Elements with Exon 6A

**Figure 4:**
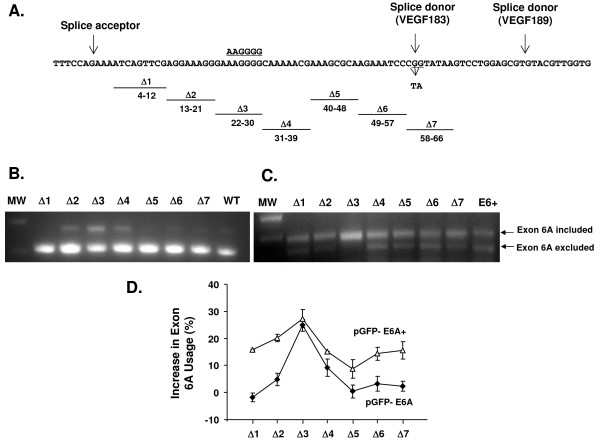
**Effect of deletions in exon 6A on splicing activity of pGFP-E6A minigenes**. **A**. Sequence of full-length human VEGF exon 6A with the splice donor and acceptor sites indicated with black arrows. The naturally occurring mutation (GG mutated to TA) in exon 6A overlapping with the splice donor for VEGF183 is shown below the sequence row. Seven sequential nine nucleotide long sequential deletions were made across VEGF exon 6A in the context of pGFP-E6A and pGFP-E6A+ plasmids, excluding the sequences near splice donor and splice acceptor sites. The putative exonic splicing silencer is shown above the sequence (underlined). **B**. RT-PCR analysis of splicing pattern 48 hr following transfection of pGFP-E6A deletion plasmids into HEK293 cells. The anticipated positions of the products from the splicing events with inclusion or exclusion of exon 6A are indicated by arrows. **C**. RT-PCR analysis of splicing pattern 48 hr following transfection of pGFP-E6A+ deletion plasmids into HEK293 cell. The anticipated positions of the products for the splicing events with inclusion or exclusion of exon 6A are indicated by arrows. MW = lane with molecular weight markers. D. The percentage of exon 6A inclusion was calculated following densitometry of the gel as [E6A included band/(E6A included band + E6A excluded band)] ×100 followed by subtraction of wildtype background. Experiments were repeated 4 times in the context of pGFP-E6A (closed symbols) or three time in the context of pGFP-E6A+ (open symbols) and the average % inclusion from repeated transfections and the standard error are shown.

We performed scanning mutagenesis across the exon 6A. Nine nucleotide long deletion mutations were made across exon 6A in the context of pGFP-E6A to locate potential splicing silencers (i.e., sequences whose deletion would result in enhanced utilization of exon 6A, Figure 4A). To identify putative exonic splicing enhancers (i.e., sequences whose deletion would result in reduced usage of exon 6A), the same deletions were introduced into the context of pGFP-E6A+, a similar plasmid with enhanced exon 6A branch point, splice acceptor and splice donor [30]. The first three nucleotides at the 5' end and the last six nucleotide at the 3' end were not mutated to avoid the disruption of normal splice site recognition. In the production of VEGF exon 6A plasmids and deletion mutants we also discovered a naturally occurring mutation in exon 6A that would mutate amino acid 133 of VEGF189 from arginine to leucine (CGG to CTA). Interestingly this mutation would disrupt the splice acceptor for VEGF183 [14]. This mutation was found to be present in 3 out of 40 alleles sequenced from blood bank samples. When this mutation was introduced into the context of the pGFP-E6A reporter plasmid, it had no impact on the usage of the exon 6A in HEK293 cells (not shown).

After transfection of the deletion mutations of pGFP-E6A into HEK293 cells, the splicing pattern was assessed by RT-PCR (Figure 4B). In the context of pGFP-E6A, Δ3 consisting of a deletion of nucleotides 22 to 30 of exon 6A, increased the inclusion of exon 6A in the mRNA by 31.7 ± 2.4 % over the native exon of transcripts contained exon 6A compared to of 6.8 ± 1.8% for the unmutated control, difference of 24.8 ± 2.1% (Figure 4D) or 4.7 ± 0.4 fold increase. The other deletions mutations had lesser impact on the inclusion of exon 6A. In the context of the pGFP-E6A+ plasmid, none of the deletion mutations resulted in enhanced exclusion of exon 6A as might be expected from deletion of an exonic splicing enhancer. But again, Δ3 promoted the maximum inclusion of exon 6A (Figure 4C), as it did in the context of the pGFP-E6A plasmid (see Figure 4D for quantitative data). This suggests the presence of an exonic splicing silencer in nucleotides 22-30 of exon 6A.

To support the experimental evidence for the existence of a possible *cis*-acting splicing silencing element, we screened the exon 6A sequence to find exonic splicing silencers (ESS) using a web based search utility http://genes.mit.edu/fas-ess/[32]. This program predicted 4 possible ESS in the sequence of exon 6A. One of these comprised the sequence AAGGGG within the 9 nucleotide deletion (AAAGGGGCA) identified experimentally.

### Mutagenesis of the Proposed Exonic Splicing Silencer

**Figure 5:**
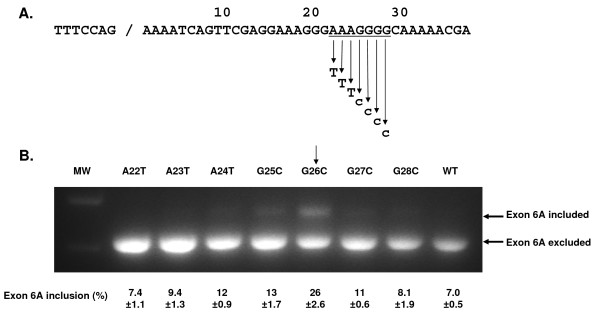
**Effect of point mutations within the VEGF exon 6A putative exonic splicing silencer**. **A**. Partial sequence of VEGF exon 6A with splice donor site and putative ESS sequence (underlined). The seven mutant versions with purine to pyrimidine transversions are shown. **B**. RT-PCR analysis of splicing pattern 48 hr following transfection of pGFP-E6A single nucleotide substitution plasmids into HEK293 cell. The anticipated positions of the products for the splicing events with inclusion or exclusion of exon 6A are indicated by arrows. MW = lane with molecular weight markers. The exon inclusion percentage was calculated following densitometry of the gel as [E6A included band/(E6A included band + E6A excluded band)] × 100. Experiment was repeated 3 times and the average % inclusion from 3 replicate transfections and the standard error was shown.

To map critical elements within the putative ESS, we created a series of single nucleotide tranversions mutants spanning the polypurine sequence (AAAGGGG) within the Δ3 deletion. These changes were introduced in the context of the pGFP-E6A minigene and the mutant reporter minigenes were transfected into HEK293 cells and the impact on splicing was assessed by RT-PCR. As observed previously, the wild type exon 6A sequence resulted in the exclusion of exon 6A but point mutations in the putative ESS resulted in increased inclusion of exon 6A. The point mutant G26C provided the maximum inclusion of exon6A (26 ± 2.6%) with the flanking mutations G25C and G27C providing a lesser inclusion level (13 ± 1.7% and 11 ± 0.6% respectively) but still greater than for the wildtype sequence (Figure 5).

The wildtype and G26C mutant sequence that promoted inclusion of exon 6A were compared with a web utility that predicts splicing factors that interact with specific sequences in pre-mRNA http://www.ebi.ac.uk/asd/[33]. The utility predicted sites where three heterogeneous nuclear riboncleoproteins (hnRNP), hnRNP F, hnRNP H, nhRNP U may interact with the nucleotides in the putative ESS [34]. Of these, the binding of hnRNP H was predicted to be eliminated by the G26C mutation.

### *In Vivo *Studies of Splicing with Deletion Mutants of Putative Exonic Splicing Silencer

**Figure 6:**
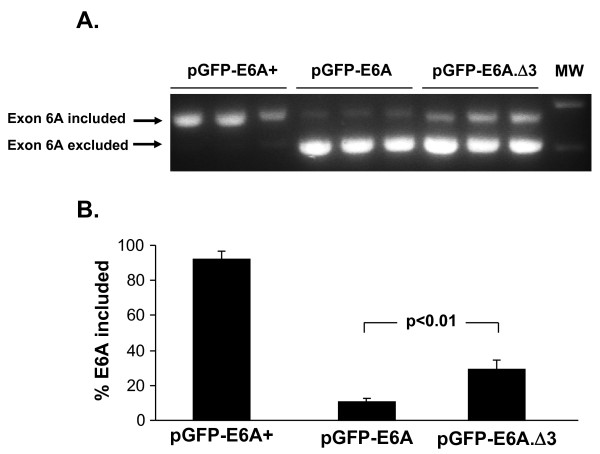
***In vivo *effects of deletion of exon 6A nucleotides 22-30 on splicing in reporter construct**. Mice (n = 3/group) were injected by high volume tail vein method with 1.6 ml of PBS containing 100 μg of plasmid. After 2 days, mice were sacrificed and liver was recovered and used for RNA preparation. Total RNA was treated with DNaseI to avoid the contamination by genomic DNA **A**. One-step RT-PCR analysis of splicing pattern. Each lane represents RT-PCR product from a different mouse. The anticipated positions of the products for the splicing events with inclusion or exclusion of exon 6A are indicated by arrows. MW = lane with molecular weight markers. **B**. The exon 6A inclusion percentage was calculated from densitometric scan of gels as described for Figure 4, and the mean ± standard deviation is plotted for each group.

Because this putative ESS was initially identified *in vitro *in a transformed cell line, we next confirmed the data *in vivo*, initially in the context of the pGFP-E6A reporter miniplasmid. High volume tail vein injection of naked plasmids was used to obtain hepatic expression of the minigene. A luciferase plasmid was coinjected to ensure that the pGFP-E6A plasmid had efficiently been delivered. RT-PCR analysis of the liver RNA showed an overall expression pattern that mimicked that seen *in vitro *(Figure 6A). For the positive control, pGFP-E6A+, 92 ± 4% of the mRNA had exon 6A included as in VEGF189. For the livers from pGFP-E6A injected mice, only 11 ± 2% of the mRNA had exon 6A included (Figure 6B). The introduction of the Δ3 mutation, deleting the putative exonic splicing silencer, resulted in 29 ± 6% of the mRNA having exon 6A included (p < 0.01 compared to pGFP-E6A group). Thus, the impact of the Δ3 mutation *in vivo *was 2.7 ± 0.3 fold enhancement of the usage of exon 6A, which is slightly less than the impact of the same mutation *in vitro*. We conclude that the putative exonic splicing silencer works similarly in the liver as it does *in vitro*, at least in the context of the minigene reporter plasmid.

**Figure 7:**
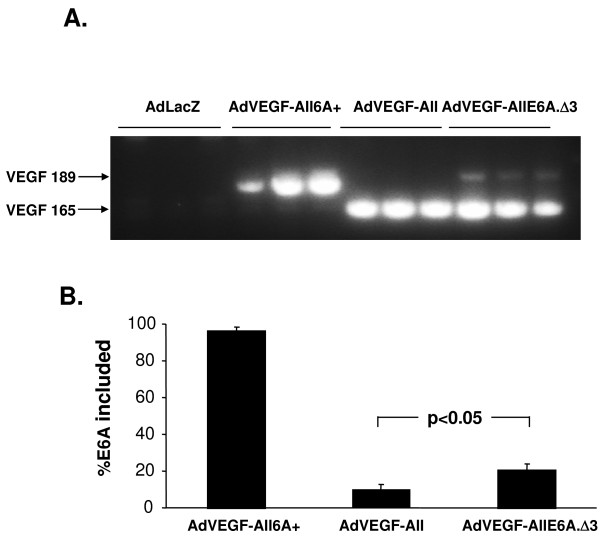
***In vivo *effects of deletion of exon 6A nucleotides 22-30 on splicing in genomic context**. Mice (n = 3/group) were administered 10^10 ^particle units of AdlacZ as negative control, AdVEGF-All, AdVEGF-All6A+ or AdVEGF-AllE6A.Δ3 by injection into the tail vein. After 2 days the mice were sacrificed and the livers were harvested. Total RNA of each liver sample was treated with DNase I. **A**. One-step RT-PCR analysis of splicing pattern. Each lane represents RT-PCR product from a different mouse. The anticipated positions of the products for the splicing events with inclusion or exclusion of exon 6A are indicated by arrows. MW = lane with molecular weight markers. **B**. The Exon6A inclusion percentage was calculated from densitometric scan of gels as described for Figure 4, and the mean ± standard deviation is plotted for each group.

Although this putative exonic splicing suppressor works well in the context of the minigene, there are differences between the minigene and the actual genomic context of the VEGF gene. The minigene contains incomplete VEGF introns 5 and 6 and, additionally, the 5' and 3' exons are derived from GFP rather than comprising VEGF exons 5 and 7. In order to confirm our results in the context of human genomic VEGF gene, the Δ3 deletion was introduced into the VEGF-All gene which consists of cDNA exons 1-5 followed by the genomic configuration of exons 6 to 8. The VEGF-AllE6A.Δ3 gene was used to make a replication deficient adenovirus vector AdVEGF-AllE6A.Δ3. This vector and the control vectors AdVEGF-All with native splicing signals and AdVEGF-All6A+ with enhanced exon 6A recognition were injected into mice by the tail vein with RT-PCR assessment of splicing pattern in liver after 2 days (Figure 7 tent with the expected splicing pattern for VEGF189. For AdVEGF-All, with the native VEGF sequences except intron 1-4, only 10 ± 3% of the RT-PCR product was consistent with the expected size for VEGF189. The introduction of the Δ3 mutation, deleting the putative exonic splicing silencer, resulted in 21 ± 4% of the RT-PCR product was consistent with the expected size for VEGF189 (p < 0.05 compared to AdVEGF-All group). These results confirm that the putative exonic splicing silencer is functional in the mouse liver in the context of the VEGF gene.

## Discussion

Assessment of the efficacy of therapeutic gene transfer with different isoforms of VEGF has demonstrated that optimum angiogenesis is achieved following delivery of a combination of VEGF isoforms with a preponderance of VEGF189 [28,30]. The optimal design of such vectors was dependent on an understanding of the *cis *and *trans *factors that dictate cell specific splicing of VEGF gene primary transcripts. However, such vectors must produce VEGF189 in tissues, such as liver, where the usual splicing pattern of the endogenous gene does not result in expression of VEGF189. In this study, we have developed a readily monitored reporter minigene construct that allows us to monitor the usage of the VEGF exon 6A, the exon used exclusively in VEGF189. The reporter minigene construct allowed identification of a putative exonic splicing silencer of exon 6A usage consisting of an oligopurine tract within exon 6A. This sequence was functional in suppressing exon 6A usage both *in vitro *and *in vivo *and was effective when introduced back into the native context of VEGF gene and assessed *in vivo*.

### Control of Splicing of the VEGF Gene

While it is clear that the different VEGF isoforms have different biological properties, there is only a rudimentary understanding of the determinants of VEGF gene splicing. However, the pattern of expression suggests complex regulation of splicing with all isoforms being expressed in a tissue, cell and developmentally controlled manner. The best described splicing determinant is the expression of the inhibitory isoform of VEGF165b for which the role of splicing factors has been assessed in cotransfection experiments [35]. The data suggest that the choice of the splice acceptor site in exon 8 is determined in part by the activity of the *trans *splicing factors 9G8 (SFRS7) and SR-55 (SFRS6). Also, the SLM-2 RNA processing protein has been shown by antisense technology to be involved with the utilization of VEGF exon 7 and expression of VEGF165 in glomerular epithelial cells [36]. Another important recent finding is the identification of factors that influence the expression of pro- and anti-angiogenic forms of VEGF based on exon 8 usage. Growth factors such as TGFβ1 result in phosphorylation of *cis *splicing factors such as SRp55 which interacts direct with the pre-RNA and influences exon 8 selection [12]. At the translational level, VEGF was also been studied for the possible regulators.

Eukaryotic initiation factor 4E (elF-4E) enhanced VEGF expression through translational regulation rather than transcriptional regulation in cells overexpressing elF-4E [37]. Prior to the current study there is no information on the mechanism by which exon 6A is selected. The lung and heart are the principal tissues that express this isoform but the critical cell-specific factors that dictate exon 6A recognition in these cells are unknown. If exon 6A, used in the formation of the mRNA for VEGF189, is a typical alternatively spliced exon, there are likely *cis *sequences in or around the exon that are recognized by cell specific factors and regulate its usage. These could either promote exon 6A usage (i.e., exonic specific enhancers) or suppress its usage (exonic specific silencers).

To identify the *cis *determinants of exon 6A usage, we used a reporter minigene system. Minigene reporter systems are commonly used to facilitate the study of alternative splicing where only one or a few exons and their flanking regions are cloned in a heterologous reporter gene context. This approach has been used in the past to assess splicing determinants in microtubule-associated protein tau exon 10, polypyrimidine tract binding protein exon 11, FGFR2 exon IIIb and IIIc, CD45 and other genes [38-44]. In this context of the minigene, exon 6A was efficiently excised, consistent with the predominance of VEGF165 over VEGF189 in most tissues and cell lines [18]. We also identified a portion of exon 6A that upon deletion lead to enhanced usage of exon 6A. The nucleotide sequence (AAAGGGGCA) overlaps with the known binding sites for three hnRNP proteins, hnRNP F, hnRNP H and hnRNP U. These and other hnRNPs are known to bind to mRNA sequences and in some contexts suppress the usage of exons to which they bind [45-49]. In particular, hnRNP H has been shown to bind to a exonic splicing silencer and control alternate splicing of the rat β-tropomyosin gene [47]. It is also involved as a negative regulator of splicing in genes such as Rous Sarcoma virus genome, bcl-x and FGFR2 genes [50-52]. The individual nucleotides of the putative exonic splicing silencer were mutated and the G26 was identified as being the most critical residues for suppression of exon 6A usage. By bioinformatic analysis, this was most consistent with the ESS being a target for hnRNP F or hnRNP H [33].

The use of a reporter minigene system in a partially transformed cells line has at least two limitations. The first is that splicing happens in an artificial context without the other VEGF introns and exons which are normally spliced sequentially and may compete for general and alternate splicing factors. The second is that the HEK293 cell line is partially transformed by the adenovirus E1 region; it is known that transformed cell lines sometimes have aberrant splicing pathways and that viral proteins have specific effects on splicing [53,54]. Therefore the ESS deletion mutant was assessed by *in vivo *gene transfer which results in expression primarily in hepatocytes [55,56]. In the minigene context *in vivo*, the deletion of the ESS had the expected effect of increasing VEGF exon 6A utilization. Therefore the deletion mutation was also reintroduced into the VEGF gene with the alternatively spliced exons in the native context [28]. In this context the deletion mutation that removed the ESS also was effective in promoting the utilization of exon 6A for expression of VEGF189.

Use of the minigene to identify a *cis*-element in VEGF exon 6A has some potential limitations. For example, the expression of minigene is not permanent so it is difficult to follow the changes in splicing events occurred during a chronic disease progression such as cancer. Also, for the GFP-minigene, differences or changes in fluorescence may not always reflect differences in splicing due to differences in transcription of the fluorescent reporter. Another limitation in our study is although we introduced the mutation of ESS back in human VEGF genomic context and showed its effect *in vivo*, the VEGF genomic sequence in this minigene lacks intron 1-4, which is still not the complete native VEGF gene. Furthermore, though our *in vitro *and *in vivo *study clearly showed the existence of ESS in VEGF exon 6A, it's also important to prove it in tissues known to express VEGF189 like the lung or the heart in future study *in vivo*.

By use of a minigene expressing a GFP reporter gene, it is possible to follow the splicing of a minigene in real time. For example, monitoring changes in splicing patterns during development as well as in the progression of disease such as cancer would be possible in transgenic mouse models. Changes in VEGF splicing have been described in embryogenic development and cancer progression[17,19,24,25,27]. There is substantial evidence that alternative splicing of the VEGF gene involved in angiogenesis can regulate the angiogenic drive in tumors, and that tumor-mediated alterations in splicing may be part of the angiogenic switch. Furthermore, drugs, such as TG003, a kinase inhibitor that targets Clk1 (Cdc2-like kinase 1) and Clk4, might be used to regulate the splicing of genes *in vitro *or/and *in vivo *[57]. The VEGF splicing reporters described here would thus be applicable for studies in these circumstances. Further development of additional reporter constructs based on the modular nature of the construct described in this paper will be useful to study of alternative splicing events in cell culture and transgenic animal models.

## Conclusion

Different isoforms of VEGF are generated by tissue specific alternate splicing and the VEGF165 and VEGF 189 isoforms differ by the presence or absence of exon 6A. In this study, a reporter minigene was used to identify *cis *sequences that regulate utilization of VEGF exon 6A and dictate the relative expression levels of VEGF isoforms. This sequence acted as a exonic splicing silencer in the context of the minigene and the native context of VEGF both *in vitro *and *in vivo*.

## Methods

### Plasmid Construction

Plasmid pAcGFP1-C1 expressing green fluorescent protein (GFP) and pAsRed2-C1 expressing red fluorescent protein (RFP) were from Clontech (Mountain View, CA). The vector pGFP-E6A, based on pAcGFP1-C1, is a GFP reporter minigene designed such that the splicing event in VEGF165 gives an active GFP protein. The VEGF exon 6A and shortened forms of its flanking introns were inserted between nucleotides 285 and 286 of the green fluorescent protein (GFP) coding sequence. The gene was assembled in three pieces. Overlap PCR was used to fuse the 5' end of the GFP gene to human VEGF intron 5 nucleotides 1 - 291. A separate PCR reaction amplified human VEGF gene from intron 5 nucleotide 1539 (274 basepairs (bp) before the splice acceptor of exon 6A), to intron 6 nucleotide 238 (with respect to VEGF189 splice donor). This domain includes exon 6A. In a third reaction, overlap PCR was used to fuse the 3' end of GFP gene to the 3' end of intron 6 of the human VEGF gene (starting 366 bp before the splice acceptor of exon 7). The length of shortened intron 5 and intron 6 were 565 bp and 604 bp respectively. The three parts were assembled and the sequence was verified (Figure 1B).

The pGFP-E6A+ plasmid was similar to pGFP-E6A except for introduction of mutations at the intron 5 branch point (from ACCTTAC to cCCTgAg) and the exon 6A splice donor (from TTTTTATTTCCAG/AA to TTTTTcTTTCCAG/AA) and exon 6A splice acceptor (from GT/GTACGT to GT/GTAaGT) to enhance usage of exon 6A[30]. Deletion mutants of pGFP-E6A and pGFP-E6A+ as well as point mutants of pGFP-E6A were also generated using QuikChange II XL Site-Directed Mutagenesis kit (Stratagene, La Jolla, CA) following the manufacturer's protocol. All mutant constructs were sequenced to confirm the presence of mutations.

### Cell Culture, Transfection and RNA Extraction

HEK293 (Human embryonic kidney cells, ATCC Manassas, VA) cells were maintained in DMEM medium at 37°C in a humidified atmosphere of 5% CO_2_. Cells (~10^6^) were transfected (Polyfect, Qiagen, Valencia, CA) with 4 μg of plasmid and harvested after 48 hr. Total RNA was prepared using RNeasy Mini kit (Qiagen) following the manufacturer's protocol.

### Reverse Transcription-PCR (RT-PCR)

RT-PCR was carried out with a one-step RT-PCR kit (Qiagen) at 50°C for 30 min, 95°C for 15 min, followed by 35 cycles of 94°C for 30 sec, 52°C for 30 sec, 72°C for 30 sec using the forward primer (5'-GCCCATCCTGATCGAGCTGAAT) and reverse primer (5'-GTGGGCGTTGTAGTTGTACTCCATCTT 3) which can specifically anneal to the 3' and 5' fragments of GFP yielding a product of 481 bp if exon 6A is utilized and 409 bp if exon 6A is excluded. To amplify mRNA expressed by the endogenous human VEGF gene in HEK293 and BT474 cell lines, RT-PCR primers consisted of the forward primer (5'-GGCCTGGAGTGTGTGCCCACTG) and reverse primer (5'-CGGCAGCGTGGTTTCTGTATCGA) which give a 477 bp product for VEGF 189, 405 bp for VEGF165 and 273 bp for VEGF121. The PCR products were separated on a 2% TBE-agarose gel, stained with ethidium bromide, and photographed on a UV transilluminator. The sequence of the RT-PCR products was confirmed by excision of the bands from the gel and DNA purification by QIAquick Gel Purification kit (Qiagen) and dideoxy sequencing. Splicing efficiency was calculated as the ratio between the intensity of the band including exon 6A divided by the sum of the intensities for the two bands including and excluding exon 6A using MetaMorph software (Universal Imaging Corporation, Downingtown, PA).

### Fluorescent Microscopy and Flow Cytometric Analysis

Cells were transfected as described above and after 48 hr were harvested using trypsin-ethylenedimane tetraacetic acid. The cells were washed twice with phosphate buffered saline (PBS), and suspended in 200 μl 2% paraformaldehyde, mixed and transferred to flow cytometry (FACS) tubes and analyzed immediately on a FACSCalibur (BD biosciences, Pharmingen). Flow cytometric data was analyzed using FlowJo software. A duplicate set of cells was analyzed by fluorescent microscopy using Olympus IX70 (Olympus Microsystems America, Redwood City, CA).

### High Volume Tail Vein Injection of Plasmids

All experiments in mice were performed under protocols approved by Institutional Animal Care and Use Committee. Male BALB/c mice (n = 3/group) received 100 μg plasmid DNA (pGFP-E6A, pGFP-E6A+, pGFP-E6Δ3) mixed with 10 μg pCMV-Luciferase diluted in PBS by tail vein injection. Mice were rapidly injected with DNA in a large volume of saline (1.6 ml) within 5 sec using a 26-gauge needle. After 48 hr, the liver of each mouse was dissected at the anterior lower part of left lobe exclusively to exclude spatial variability, snap-frozen in dry ice and stored at -80°C until further processing. Luciferase assay of liver homogenate was used to verify the success of the high volume tail vein injection method. For mouse tissue samples, the RNeasy Midi kit (Qiagen) was used to isolate the total RNA. The RNA samples were then treated with DNase I (Invitrogen, Carlsbad, CA) to remove genomic DNA contaminants and then subjected to RT-PCR as described above.

### Adenovirus Vector Construction

VEGF-All is a hybrid cDNA/genomic gene comprising the human VEGF cDNA exons 1-5 followed by the genomic configuration of exons 6 to 8 with the exception of an internal deletion to reduce the size of intron 7 from 2425 to 721 bp. AdVEGF-All is an E1^- ^E3^- ^adenovirus (Ad) vector expressing VEGF-All from the cytomegalovirus immediate early promoter/enhancer (CMV) [30]. AdVEGF-All6A+ is similar to AdVEGF-All except for three mutations in the splicing signals around exon 6A that promote inclusion of exon6A in the mRNA. The AdVEGF-AllE6A.Δ3 is identical to AdVEGF-All except the nucleotides 22-30 of exon 6A were deleted. A similar Ad vector with LacZ transgene (AdLacZ) was used as a negative control. Vectors were propagated on HEK293 cells and purified by two cesium chloride density gradients. Dosing was carried out based on particle units (pu).

### *In Vivo *Splicing of VEGF Exon 6A Expressed from Adenoviral Vectors

For *in vivo *experiments, 6 to 8 wk BALB/c mice were injected through the tail vein with 10^10 ^pu of AdVEGF-All, AdVEGF-All6A+ or AdVEGF-AllE6A.Δ3 in 100 μl PBS. After 48 hr, the liver of each mouse was collected for RNA isolation and analysis by RT-PCR with forward primer located in exon 3 and exon 4 (5'-ATCACCATGCAGATTATGCGGATC) and a reverse primer (5'-GTGGTATGGCTGATTATGATCAG) located in the polyA site of the CMV based plsmid so that only the splicing variants of the adenovirus-minigene could be amplified and the endogenous VEGF will not be picked up.

### Statistical Analysis

To quantitatively assess differences in splicing patterns among different groups, digital photographs of stained gels were quantified by Metamorph software adjusting for background in each lane. The percentage of the total intensity attributable to VEGF189 was compared for different groups by two tailed Student's t test.

## Authors' contributions

RW carried out the *in vitro *and *in vivo *experimental work. RGC provided assistance with experiment design, interpretation and the scientific context of the work performed. NRH took overall responsibility for the project, data analysis and writing up the article. All authors read and approved the final version of the manuscript.
